# Amyloid beta emerges from below the neck to disable the brain

**DOI:** 10.1371/journal.pbio.3001388

**Published:** 2021-09-15

**Authors:** Grant Kauwe, Tara E. Tracy

**Affiliations:** Buck Institute for Research on Aging, Novato, California, United States of America

## Abstract

Accumulation of amyloid beta (Aβ) in the brain in Alzheimer disease drives pathophysiology. A study in this issue of *PLOS Biology* revealed that Aβ from the liver can promote brain pathology, supporting that peripheral Aβ can contribute to neurodegeneration.

Alzheimer disease is the most common type of dementia, and it is characterized by the presence of amyloid plaques in the brain that are comprised of aggregated amyloid beta (Aβ) peptides. The Aβ peptide is a fragment of the larger integral membrane protein called amyloid precursor protein (APP) that is expressed in neurons of the brain. When APP is cleaved by β and ɣ secretase enzymes, the resulting Aβ peptides form small soluble aggregates that induce toxicity in the brain and neuronal dysfunction followed by the deposition of Aβ peptides into insoluble amyloid plaques. Many studies have demonstrated that transgenic mice with expression of human APP carrying Alzheimer disease–causing mutations in the brain have high levels of toxic Aβ, neuronal pathophysiology, memory impairments, and plaque formation [1]. The deleterious impact of Aβ produced inside the brain related to memory loss and Alzheimer disease is clear, but Aβ can also be detected outside of the central nervous system in the blood where it has growing potential as an effective biomarker for Alzheimer disease [2]. The soluble Aβ detected in blood could be peptides that are cleared into the blood from the brain [3], but Aβ can also originate from cells in the periphery. Whether peripheral production of Aβ can contribute to Alzheimer disease pathogenesis is not clear. Now, Lam and colleagues have generated a new transgenic mouse with expression of human mutant APP primarily in the liver to investigate whether peripheral production of Aβ is sufficient to promote neurodegenerative phenotypes in the brain (Fig 1) [4].

**Fig 1:**
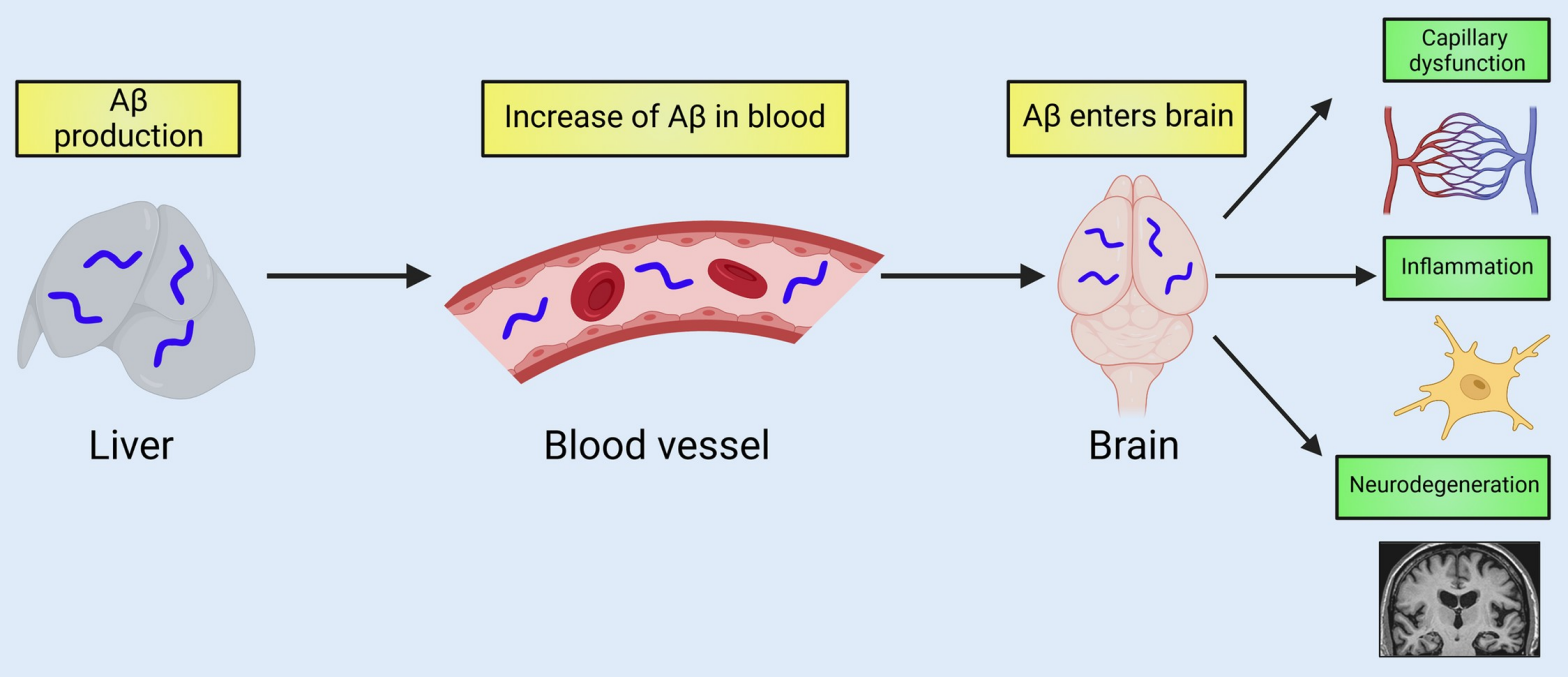
Aβ produced in the liver contributes to disease-related pathology in the brain. Lam and colleagues generated HSHA transgenic mice that produce pathogenic Aβ specifically in the liver. Liver-derived Aβ travels through the circulatory system and crosses the blood–brain barrier to enter the brain. Aβ accumulation in the brain drives Alzheimer disease–related pathology including capillary dysfunction, inflammation, and neurodegeneration. Created with BioRender.com. Aβ, amyloid beta; HSHA, hepatocyte-specific human amyloid.

Higher levels of Aβ peptide isoforms were found in the blood of individuals with pathological Aβ accumulation in brain and cognitive impairment [2]. This correlative increase in Aβ could be due to the transfer of brain-derived Aβ across the blood–brain barrier as a mechanism of Aβ clearance. On the other hand, Aβ found in blood is associated with lipoprotein particles, and various reports provide evidence that peripheral lipogenic organs could be another source of Aβ peptides in the blood [5,6]. However, given the robust accumulation and effect of Aβ originating in the central nervous system, the potential impact of peripheral Aβ on the brain in disease etiology is often overlooked. Lam and colleagues sought to address this gap in knowledge in the neurodegeneration field by elucidating the effect of peripheral Aβ on the brain. They created a knock-in mouse for Cre recombinase–dependent expression of human APP carrying the familial Alzheimer disease Swedish (KM670/671NL) and Indiana (V717F) mutations and bred it with a previously established liver cell–specific Alb-Cre mouse line [7] to make hepatocyte-specific human amyloid (HSHA) mice. An important consideration when using a cell-specific Cre mouse line such as this is that unexpected transient expression of Cre recombinase may occur in other cell types or tissues. Importantly, the authors confirmed specificity by detection of high levels of mRNA for the human APP transgene in the liver of HSHA mice, with no significant detection of mRNA for the transgene in the brain, duodenum, or lung. Despite the apparent lack of APP transgene expression in the brain during aging, positron emission tomography (PET) imaging in vivo showed that the HSHA mice had an age-dependent increase in Aβ deposition in the brain. This suggests that human Aβ produced in the liver can enter the brain. Notably, more than a decade ago, researchers showed that Aβ administration into the periphery by intraperitoneal injection causes β-amyloidosis in the brain [8], yet how Aβ is transported across the blood–brain barrier and whether it involves the association of Aβ with lipoproteins or a breakdown of the blood–brain barrier remain unclear.

Commonly used transgenic mouse models with human APP transgenes carrying familial mutations that produce high Aβ levels in the central nervous system recapitulate important features of Alzheimer disease. Studies on these mice have long served as a foundation for the advancement of new therapies. The age-dependent pathological phenotypes reported in the HSHA mice created by Lam and colleagues include abnormal lipid accumulation in the brain, neurodegeneration, and the dysfunction of capillaries associated with lipofuscin aggregation. The HSHA mice also demonstrated impaired performance in the passive avoidance test of hippocampal-dependent memory at 12 months of age. Other human APP mouse models with Aβ produced in the brain can have more aggressive Alzheimer disease–related pathology and earlier onset cognitive impairments in a range of behavioral tests. It is important to consider that the extent of the pathological effect of peripheral Aβ on the brain may depend on a limited amount of Aβ that enters the brain and the region where it accumulates upon entry. Nevertheless, this new study supports that peripheral Aβ is sufficient to promote pathological disease–associated features in the brain and memory impairment.

Could targeting peripheral Aβ be another strategy to prevent neurodegeneration? Pharmacological reduction of Aβ levels in the blood of wild-type mice by peripheral administration of a drug impermeable to the blood–brain barrier was linked to reduced Aβ levels in the brain [9]. Dietary fatty acids can also modulate plasma Aβ levels in wild-type mice [10]. These findings provide evidence that Aβ levels in blood can be modulated; however, further studies are needed to establish whether or not peripherally targeted strategies to reduce Aβ would be beneficial for brain function and cognition in the context of Alzheimer disease. Historically, many different approaches have been explored to try to reduce Aβ levels in the brain as a disease-modifying therapy for Alzheimer disease [11]. However, numerous clinical trials on therapies targeting Aβ have failed and did not meet expectations to slow cognitive decline in patients with symptomatic Alzheimer disease. This obstacle to finding an effective disease-modifying treatment for Alzheimer disease could be attributed to the complexity of the mechanisms that contribute to the disease progression that involves multiple pathologies [12]. Thus, lowering Aβ levels in combination with targeting other pathogenic factors in Alzheimer disease may be necessary to ameliorate cognitive decline. It is also possible that therapies directed against peripheral Aβ in addition to Aβ produced in the brain could serve to further dampen amyloid toxicity in the brain. The HSHA transgenic mice created and characterized by Lam and colleagues in this report could be used to further interrogate the therapeutic potential of targeting peripheral Aβ production to reduce the risk of Alzheimer disease.

## Abbreviations

Aβ: amyloid beta
APP: amyloid precursor protein
HSHA: hepatocyte-specific human amyloid
PET: positron emission tomography
